# Limitations of photodynamic therapy in HPV-positive Erythroplasia of Queyrat: A case of progression to carcinoma in situ

**DOI:** 10.1097/MD.0000000000045706

**Published:** 2025-10-31

**Authors:** Yangfeng Lou, Qiuyan Duan, Longfei Yang, Peng Zhou

**Affiliations:** a Department of Urology, Hangzhou Third People’s Hospital, Hangzhou, China; b Department of Dermatology, Hangzhou Third People’s Hospital, Hangzhou, China.

**Keywords:** Erythroplasia of Queyrat, HPV type 18, human papillomavirus, penile carcinoma in situ, photodynamic therapy, risk stratification

## Abstract

**Rationale:**

This case of Erythroplasia of Queyrat (EQ) in a 52-year-old male, driven by high-risk human papillomavirus (HPV)-18, is notable for progressing to carcinoma in situ despite photodynamic therapy (PDT). It highlights PDT’s limitations in HPV-positive EQ, emphasizing HPV genotyping and aggressive initial therapies to prevent malignancy.

**Patient Concerns:**

The patient had a persistent, erythematous, velvety plaque on the glans penis with mild pruritus. Examination showed a well-demarcated lesion.

**Diagnoses:**

Biopsy confirmed dysplastic squamous epithelium. Molecular testing identified HPV-18, indicating high malignant potential, leading to a diagnosis of EQ.

**Interventions:**

The patient received PDT, followed by conservative treatments including topical therapies upon recurrence.

**Outcomes:**

PDT achieved partial lesion regression, but recurrence as carcinoma in situ was confirmed histologically. Conservative treatments failed to stop progression, with HPV-18 driving the aggressive course, necessitating escalated interventions.

**Lessons:**

HPV genotyping is vital to identify high-risk subtypes like HPV-18; PDT may be inadequate for HPV-positive EQ, requiring aggressive therapies; rigorous long-term surveillance is essential to prevent squamous cell carcinoma progression.

## 1. Introduction

Erythroplasia of Queyrat (EQ) is one of the rarest but most aggressive premalignant entities with a predilection for the glans penis with a high likelihood of progression to clinically invasive squamous cell carcinoma. A case report describing a 52-year-old man with EQ who thereafter developed carcinoma in situ (CIS) after treatment by photodynamic therapy (PDT). While the patient did, at first, respond to PDT, they reacted poorly as subsequent disease progression was explained by high-risk human papillomavirus (HPV) type 18 infection. Significant etiological limitations of PDT include the superficial depth of penetration, which is only 2 mm, and the difficulty in achieving homogeneous delivery on inhomogeneous surfaces, both of which are important causes of treatment failure. Additionally, HPV oncogenic mechanisms, such as immune escape through reduced major histocompatibility complex I expression and the secretion of immunosuppressive cytokines, further exacerbate treatment. This analysis highlights the necessity of HPV testing in high-risk patients, the importance of more aggressive upfront treatment approaches and vigilant surveillance for disease progression.

## 2. Case presentation

A 52-year-old male patient presented with a persistent erythematous lesion on the glans penis, consistent with EQ. Clinical parameters included a body mass index of 24.5 kg/m², blood pressure of 128/82 mm Hg, heart rate of 76 bpm, and no signs of systemic involvement such as lymphadenopathy or distant metastases upon physical examination. Routine blood tests were performed to assess overall health and rule out comorbidities; results are summarized in Table 1.

**Table 1:** Clinical parameters of the patient.

Parameter	Value	Reference range
Hemoglobin (g/dL)	14.2	13.5–17.5
White blood cell count (×10^9^/L)	6.8	4.0–11.0
Platelet count (×10^9^/L)	220	150–450
Erythrocyte sedimentation rate (mm/h)	12	0–20
C-reactive protein (mg/L)	2.5	<10.0
Fasting blood glucose (mmol/L)	5.4	3.9–6.1
Creatinine (µmol/L)	85	60–110
Alanine aminotransferase (U/L)	28	7–56
Aspartate aminotransferase (U/L)	25	8–48
HIV serology	Negative	Negative

HIV = human immunodeficiency virus.

The patient’s medical history was notable for a 30-pack-year smoking history, occasional alcohol consumption (approximately 10 units/wk), and a prior diagnosis of genital warts treated with cryotherapy 5 years ago. He reported no history of immunosuppression, diabetes, or other malignancies. No sexually transmitted infections were documented in the past decade, aside from the resolved warts. Family history was unremarkable, with no reported cases of penile cancer, HPV-related malignancies, or hereditary syndromes among first-degree relatives.

**Figure 1.**
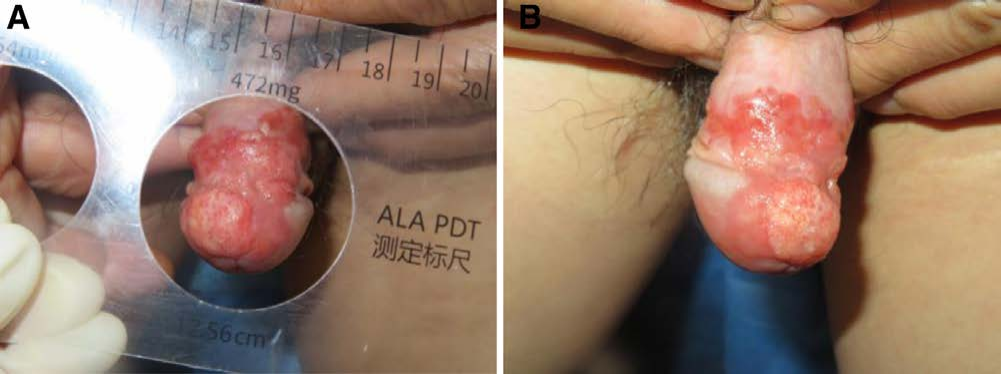
The lesion presented as a well-demarcated, erythematous, moist, velvety plaque measuring 1.5 × 1.0 cm on the dorsal aspect of the glans penis.

A well-defined, slightly elevated erythematous plaque (1.5 × 1.0 cm) was found on the glabrous surface of the glans penis after the physical examination (Fig. 1). Tender, erythematous ulcerating lesion. Through core needle biopsy, we confirmed the initial diagnosis of EQ. I discussed treatment options with the patient, and he chose PDT because of its conservative and favorable cosmetic outcomes.

**Figure 2.**
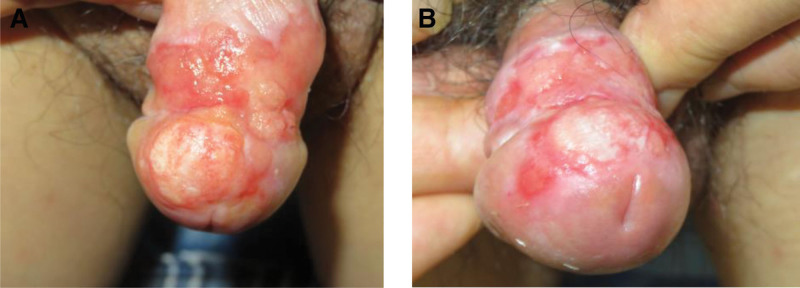
Following 2 sessions of PDT, clinical examination demonstrated significant regression of the lesion with marked reduction in both size and erythema of the original plaque. PDT = photodynamic therapy.

**Figure 3.**
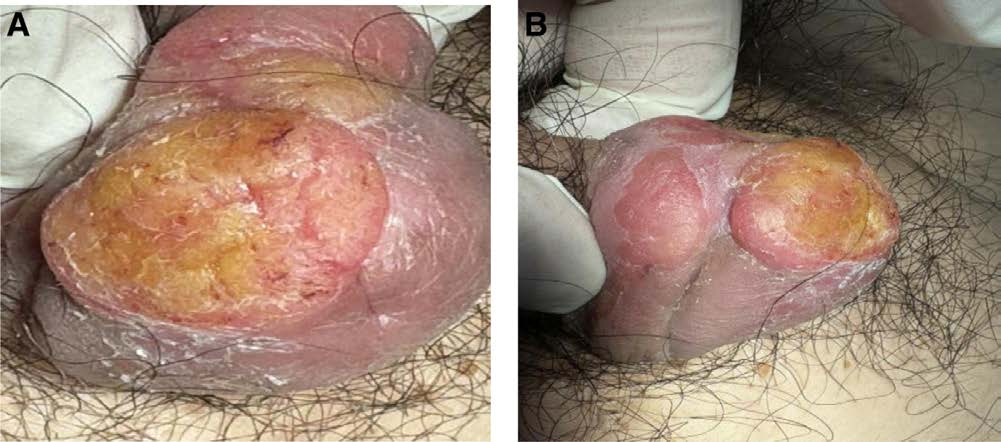
Clinical examination at 2 months revealed disease progression with the erythematous lesion expanding to 2.5 × 2.0 cm, larger than the initial presentation.

**Figure 4.**
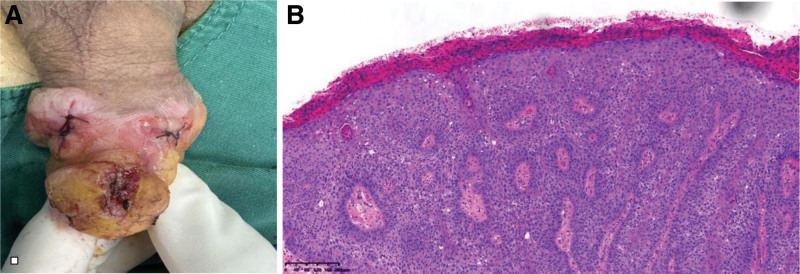
Multiple punch biopsies from different areas of the lesion revealed extensive epidermal atypia and dedifferentiation consistent with carcinoma in situ throughout all sampled sites.

**Figure 5.**
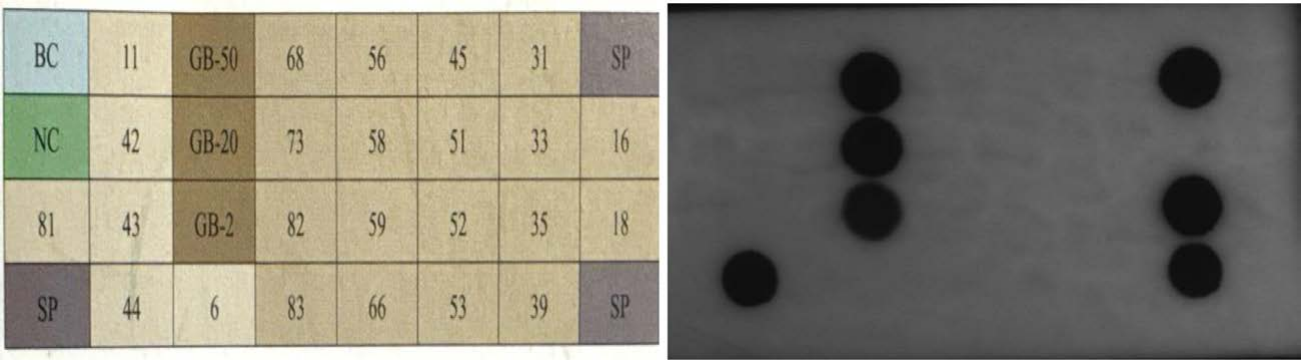
HPV genotyping using reverse dot blot hybridization with multiple control probes (quality control, internal control, positive, and negative controls) demonstrated positive detection of HPV type 18. HPV = human papillomavirus.

The treatment regimen consisted of 2 PDT sessions, separated by a 3-week interval. Application of 5-aminolevulinic acid followed by a 3-hour incubation period was performed, and then red-light illumination (630 nm, 37 J/cm^2^) was administered. Following the first 2 PDT sessions, a remarkable response was observed, characterized by a substantial reduction in lesion size and erythema (Fig. 2). Nonetheless, on the follow-up visit up to 2 months later, the size of the erythematous patch had increased to 2.5 × 2.0 cm in extent (Fig. 3). Based on this alarming progression, we underwent an incisional biopsy. The histopathologic exam showed extensive epidermal atypia and dysdifferentiation indicative of CIS (Fig. 4). Immunohistochemical staining reported diffuse p16 overexpression and high Ki-67 expression, indicative of aggressive disease progression. Using the HPV nucleic acid detection kit (biochip method) with the Bo-hui Company’s (Beijing, China) BWHF-VI nucleic acid chip detector, follow-up molecular analysis and HPV DNA typing revealed infection with HPV type 18, a high-risk genotype associated with increased oncogenic potential (Fig. 5). The patient underwent partial penile resection surgery and recovered well.

## 3. Discussion

EQ, a high-risk condition based on accurate high-risk HPV infections like HPV16 and HPV18, is ultimately CIS of the penis. PDT is an effective, minimally invasive tissue-preserving therapy for EQ. The selective accumulation of photosensitizers in dysplastic cells is followed by the specific wavelength light activation, which induces the generation of reactive oxygen species and leads to PDT, ultimately resulting in apoptosis and necrosis in pancreatic ductal adenocarcinoma. PDT also triggers a local immune response, which contributes to the tumor’s regression.

Response rates range from 50% to 80%^[1,2]^ in cervical intraepithelial neoplasia despite PDT’s reported efficacy; however, this therapy has inherent limitations. Both the photosensitizer and activating light rarely penetrate deeper than 2 mm,^[3]^ with the penetration depth often being constrained by cell density or diffusion limitation, so a large portion of the dysplastic tissue could be surrounded without adequate superficial illumination. If it weren't for the complex anatomy of the glans penis—which is far from a uniform surface—and its unpredictable blood vessels (often hidden or embedded within), it would be easier to achieve even distribution of the photosensitizer and consistent light delivery. More profound molecular disruptions caused by aberrant oncoproteins of high-risk HPV can also significantly influence treatment efficacy.^[4]^

Our case’s initial positive response PDT was followed by rapid lesion progression, exposing key limitations. PDT’s shallow penetration depth restricts its effectiveness to superficial lesions, leaving malignant cells beyond the 2 mm threshold untreated. The irregular dermis and vascularized surface of the glans penis further complicate uniform photosensitizer absorption and illumination, increasing the risk of incomplete treatment.^[5]^ High-risk HPV 18 infection contributed to treatment failure, as the anatomical irregularity and vascularization of the glans penis impaired accurate dosimetry and delivery. HPV oncoproteins also modulate the tumor microenvironment, evading immune detection by altering major histocompatibility complex class I antigen expression and secreting immunosuppressive cytokines, such as IL-10, which hinder dendritic cell maturation and T-cell activation.^[6,7]^

Immunohistochemical analysis revealed strong p16 positivity and high Ki-67 expression, both of which are associated with aggressive lesion behavior. p16 positivity, a marker for high-risk HPV infection, correlates with increased progression risk, while elevated Ki-67 indicates heightened proliferative activity and a poorer prognosis (*P* < .001).^[8,9]^ These findings underscore the need for risk stratification and the development of alternative or combined therapeutic approaches for patients at high risk of adverse outcomes.

EQ management requires thorough risk stratification before treatment planning, with high-risk features including lesion size >1.5 cm, HPV 16/18 infection, aggressive p16 and Ki-67 expression, and prior treatment failure. A comprehensive genital examination, incisional biopsy, HPV typing, and molecular marker analysis are essential for guiding personalized treatment strategies.^[10,11]^ While PDT remains a tissue-sparing option, its limitations necessitate the use of alternative or combined therapies. Surgical interventions, such as wide local excision or glans resurfacing, are effective for high-risk lesions.^[12]^ Combining PDT with topical Imiquimod or checkpoint inhibitors (e.g., PD-1 antibodies) may enhance immune response and counter evasion.^[13]^ Patients should be informed of PDT’s failure rate (10–30%) and monitored closely for recurrence.^[14]^

Hypericin-photodynamic therapy (H-PDT) is an improvement over the conventional PDT for high-risk EQ patients.^[2]^ Targeting penetration depth and treatment resistance: high-power light sources and progressive transformers for optimized photosensitizers (5-aminolevulinic acid methyl ester) are used in Hypericin-PDT to overcome the limitations of these approaches. It allows the light beam with high intensity to penetrate deeper layers of tissue and significantly affect malignant cells outside the superficial layers. Furthermore, it induces a heat shock response that increases the number of reactive oxygen species, not only causing apoptosis in the tumor cell itself but also ill itself but also in the tumor microenvironment, including its vasculature.^[1,2]^

### 3.1. Future directions

A structured follow-up protocol is crucial for effective EQ management. In the first 3 months, weekly clinical examinations with photographic documentation are recommended to monitor treatment response. Monthly evaluations are advised from 3 to 12 months, with molecular marker assessments every quarter, particularly during the 4th and 12th months. Beyond 12 months, biannual clinical evaluations and annual HPV testing are essential for long-term surveillance.^[15]^ Immediate intervention is necessary if rapid lesion enlargement, new ulcers, bleeding, or increasing pain are observed. Molecular marker analysis plays a key role, and significant marker decline or early relapse (e.g., within 2 intervals) may justify modifying the treatment plan. This approach ensures timely detection of recurrence and improves patient outcomes.^[16]^

The limitations of photodynamic therapy in high-risk EQ patients necessitate innovative, integrated therapeutic strategies. Future investigations into Hypericin-PDT for HPV-positive EQ should prioritize synergistic approaches, including: integration of Hypericin -PDT-induced immunogenic cell death with immune checkpoint inhibitors (e.g., anti-PD-1/anti-PD-L1) to potentiate antitumor immunity via tumor-associated antigens and T-cell priming^[2–4,6,17]^; combination with HPV-specific vaccines (targeting E6/E7 oncoproteins in HPV16/18) to enhance viral cell recognition and eradication^[3,4,17]^; CRISPR-Cas9-mediated editing to disrupt HPV oncogenes (E6/E7), thereby preventing progression and recurrence while addressing molecular etiology^[3]^; nanoparticle-based delivery systems to optimize photosensitizer specificity, bioavailability, and co-administration of immunomodulators or chemotherapeutics, overcoming penetration depth constraints and enabling multisite targeting; and adjunctive antiviral agents (e.g., nucleoside analogs) to augment management of HPV-associated lesions.

### 3.2. Diagnostic approach and avoidance of misdiagnosis

Given EQ’s high misdiagnosis risk due to resemblance to benign/inflammatory dermatoses (e.g., psoriasis, chronic dermatitis),^[18]^ diagnostic precision was achieved via: early incisional biopsy with histopathology (HE staining, HPV-16/18 Immunohistochemistry) confirming full-thickness epidermal atypia; PCR for high-risk HPV subtypes to exclude non-HPV lesions; detailed clinical assessment of EQ hallmarks (demarcated plaques, persistent erosions) plus risk factors (age, sexual history); multidisciplinary review by dermatology, pathology, and oncology to minimize bias; and dermoscopy for border evaluation, ruling out invasion. This approach ensured accurate diagnosis, timely photodynamic therapy, and a replicable model for high-risk cases, boosting clinician confidence and outcomes.

## 4. Conclusion

Achieving long-term success in EQ management will require a multidisciplinary approach that integrates advanced diagnostics, personalized treatment planning, and rigorous follow-up protocols to ensure tailored and effective care for each patient.

## Author contributions

**Data curation:** Longfei Yang.

**Investigation:** Yangfeng Lou, Qiuyan Duan.

**Supervision:** Peng Zhou.

**Visualization:** Qiuyan Duan.

**Writing – original draft:** Yangfeng Lou.

**Writing – review & editing:** Peng Zhou.
